# Epigenetic regulation of rice flowering and reproduction

**DOI:** 10.3389/fpls.2014.00803

**Published:** 2015-01-28

**Authors:** Jinlei Shi, Aiwu Dong, Wen-Hui Shen

**Affiliations:** ^1^State Key Laboratory of Genetic Engineering, Collaborative Innovation Center of Genetics and Development, International Associated Laboratory of CNRS-Fudan-HUNAU on Plant Epigenome Research, Department of Biochemistry, Institute of Plant Biology, School of Life Sciences, Fudan UniversityShanghai, China; ^2^CNRS, Institut de Biologie Moléculaire des Plantes, Université de StrasbourgStrasbourg, France

**Keywords:** chromatin, epigenetics, flowering time, histone modification, DNA methylation, non-coding RNA, reproduction, Oryza sativa

## Abstract

Current understanding of the epigenetic regulator roles in plant growth and development has largely derived from studies in the dicotyledonous model plant *Arabidopsis thaliana*. Rice (*Oryza sativa*) is one of the most important food crops in the world and has more recently becoming a monocotyledonous model plant in functional genomics research. During the past few years, an increasing number of studies have reported the impact of DNA methylation, non-coding RNAs and histone modifications on transcription regulation, flowering time control, and reproduction in rice. Here, we review these studies to provide an updated complete view about chromatin modifiers characterized in rice and in particular on their roles in epigenetic regulation of flowering time, reproduction, and seed development.

## INTRODUCTION

Epigenetics is defined as nucleotide sequence-independent changes in the gene expression that are mitotically and/or meiotically heritable. The fundamental repeating unit of chromatin is nucleosome. The nucleosome contains 145–147 base pairs (bp) of DNA wrapped around an octamer of histone proteins, comprising two copies of each of the four core histones, H2A, H2B, H3, and H4 (McGinty and Tan, 2014). The linker histone H1 associates with DNA inbetween the two nucleosomes and participates in higher order chromatin structure formation and remodeling. The structure of chromatin can be subjected to panoply of epigenetic regulations including DNA methylation, histone covalent modifications, histone variants, and ATP-dependent chromatin remodeling. DNA methylation has been widely considered as a heritable epigenetic mark that regulates expression of genes in both plants and mammals (Law and Jacobsen, 2010; Furner and Matzke, 2011; Wu and Zhang, 2014). Histone modifications including methylation, acetylation, phosphorylation, ubiquitination, and sumoylation, play critical roles in regulating chromatin structure and gene expression, mainly by altering nucleosome stability and positioning that affect DNA accessibility for regulatory proteins or protein complexes involved in transcription, DNA replication and repair (Pikaard and Scheid, 2013; To and Kim, 2014; Van Lijsebettens and Grasser, 2014). ATP-dependent chromatin remodeling factors control relocation or dissociation of nucleosomes, and histone chaperones bind histones and play crucial roles in nucleosome assembly/disassembly in diverse chromatin metabolism and epigenetic regulation (Zhu et al., 2012; Gentry and Hennig, 2014).

Rice (*Oryza sativa* ) is a worldwide crop and represents a valuable model plant for monocots, to which many of our food crops belong. Compared to the extensively studied dicot model plant *Arabidopsis thaliana*, rice has only been more recently studied in epigenetic modifications (reviewed in Chen and Zhou, 2013). Genome-wide analyses of DNA methylations have revealed conservation as well as distinct differences between rice and *Arabidopsis*, and that a much higher level of DNA methylation is observed in association with more numerous transposable elements present in the rice genome (Yan et al., 2010; Zemach et al., 2010; Chodavarapu et al., 2012; Li et al., 2012). Genome-wide analyses by chromatin immunoprecipitation combined with high-throughput sequencing (ChIP-Seq) have shown that several types of histone modifications, e.g., histone H3 lysine 9 acetylation (H3K9ac) and H4K12ac, H3K4 di-/tri-methylation (H3K4me2/3), H3K27me3, and H3K36me3, are broadly distributed with distinct patterns within the rice genome (He et al., 2010; Malone et al., 2011; Du et al., 2013). In this review, we summarize and discuss regulators involved in different types of chromatin modifications and their roles in rice plant flowering time control and reproduction.

## REGULATION OF DIFFERENT TYPES OF CHROMATIN MODIFICATIONS IN RICE

Different types of chromatin modifications are regulated by specific factors that are generally conserved in rice and other plant species (www.chromdb.org). So far, only some of the rice chromatin modifiers are functionally characterized by analysis of loss-of-function mutants and RNAi or overexpression transgenic plants (**Table 1**).

**Table 1:** Chromatin modifiers functionally characterized in rice.

	Name	Gene locus	Molecularfunction	Biological role	Reference
DNA methylation	OsMET1b/OsMET1-2	LOC_Os07g08500	DNA methyltransferase	Seed development	Hu et al. (2014), Yamauchi et al. (2014)
	OsDRM2	LOC_Os03g02010	*De novo* DNA methyltransferase	Pleiotrpic effects on development	Moritoh et al. (2012), Pang et al. (2013)
	OsDDM1	LOC_Os09g27060	DNA methylation maintenance	Transposon repression, growth inhibition	Higo et al. (2012)
DNA demethylation	OsROS1a	LOC_Os01g11900	DNA demethylase	Plant reproduction	Zemach et al. (2010), Ono et al. (2012)
	OsROS1c	LOC_Os05g37350	DNA demethylase	Transposon activation	La et al. (2011)
Histone methylation	SDG714	LOC_Os01g70220	H3K9 methyltransferase	Transposon repression, trichome development	Ding et al. (2007b)
	SDG728	LOC_Os05g41170	H3K9 methyltransferase	Transposon repression, seed development	Qin et al. (2010)
	SDG725	LOC_Os02g34850	H3K36 methyltransferase	Hormone regulatory gene activation, flowering	Sui et al. (2012, 2013)
	SDG724	LOC_Os09gl3740	H3K36 methyltransferase	Flowering	Sun et al. (2012)
	SDG723/OsTrx1	LOC_Os09g04890	H3K4 methyltransferase	Flowering	Choi et al. (2014)
Histone demethylation	JMJ706	LOC_Os10g42690	H3K9 demethylase	Floral organ development	Sun and Zhou (2008)
	
	JMJ705	LOC_Os01g67970	H3K27 demethylase	Biotic stress response, plant reproduction	Li et al. (2013)
	JMJ703	LOC_Os05g10770	H3K4 demethylase	Stem elongation, transposon repression	Chen et al. (2013); Cui et al. (2013)
	JMJ701	LOC_Os03g05680	H3K4 demethylase	Flowering	Yokoo et al. (2014)
Polycomb silencing	OsiEZ1/SDG718	LOC_Os03g19480	H3K27 methyltransferase	Flowering	Liu et al. (2014)
	
	OsCLF/SDG711	LOC_Os06gl6390	H3K27 methyltransferase	Flowering	Liu et al. (2014)
	OsFIE1	LOC_Os08g04290	*Drosophila* ESC homolog	Pleiotrpic effects on development	Zhang et al. (2012b), Nallamilli et al. (2013), Folsom et al. (2014)
	OsFIE2	LOC_Os08g04270	*Drosophila* ESC homolog	Organ generation, reproduction	Luo et al. (2009), Li et al. (2014)
	OsEMF2b	LOC_Os09g13630	*Drosophila* Su(z)12 homolog	Floral organ development	Yang et al. (2013), Conrad et al. (2014)
Histone deacetylation	OsHDT1/HDT701	LOC_Os05g51840	H4 deacetylase	Biotic stress response, heterosis	Li et al. (2011a), Ding et al. (2012a)
	OsSRT1	LOC_Os04g20270	H3K9 deacetylase	Cell death, transposon repression	Huang et al. (2007), Zhong et al. (2013)
Others	CHD3/CHR729	LOC_Os07g31450	Chromodomain and PHD-domain protein	Pleiotrpic effects on development	Hu et al. (2012)
	MEL1	LOC_Os03g58600	AGO-family protein	Meiosis progression	Nonomura et al. (2007), Komiya et al. (2014)
	SHO1	LOC_Os04g43050	Homolog of DICER-LIKE 4	Pleiotrpic effects on development	Abe et al. (2010)
	SHL2	LOC_Os01g34350	RDR6 homolog	Floral organ development	Toriba et al. (2010)
	WAF1	LOC_Os07g06970	HEN1 homolog	Pleiotrpic effects on development	Abe et al. (2010)
	BRK1	LOC_Os07g32480	H2A phosphorylation	Meiosis progression	Wang et al. (2012)

### DNA METHYLATION

In plants, DNA methylation occurs at cytosine residues in symmetric, CG and CHG, as well as asymmetric, CHH, contexts (where H = A, T or C; Law and Jacobsen, 2010). In *Arabidopsis*, CG methylation is maintained by METHYLTRANSFERASE 1 (MET1; Saze et al., 2003), whereas CHG methylation is mediated by CHROMOMETHYLASE 3 (CMT3; Lindroth et al., 2001). The maintenance of CHH methylation is carried out by CMT2 and DOMAINS REARRANGED METHYLTRANSFERASE 2 (DRM2), an ortholog of mammalian Dnmt3 (Law and Jacobsen, 2010; Stroud et al., 2014). DRM2 is required for *de novo* cytosine methylation in both symmetric and asymmetric sequence contexts, which is guided to the target region by RNA-directed DNA methylation (RdDM) pathway (Cao and Jacobsen, 2002; Law and Jacobsen, 2010; Stroud et al., 2014). While *Arabidopsis* contains only one *MET1* gene, rice has two *MET1* genes, *MET1a* (also named *OsMET1-1*) and *MET1b/OsMET1-2* (Teerawanichpan et al., 2004; Yamauchi et al., 2008). The transcripts of *MET1b* accumulate more abundantly than those of *MET1a* in all of the examined rice tissues, indicating that *MET1b* may play a more important role in maintaining DNA methylation (Yamauchi et al., 2008). Consistently, more recent studies demonstrate that *MET1b* is an essential gene and its loss causes genome-wide reduction of CG methylation in rice seedlings (Hu et al., 2014; Yamauchi et al., 2014). Rice contains also one *DRM2* gene, *OsDRM2*, and the recombinant OsDRM2 protein expressed in *Escherichia coli* or *Saccharomyces cerevisiae* exhibits stochastic *de novo* DNA methyltransferase activity *in vitro* at CG, CHG, and CHH (Sharma et al., 2009; Pang et al., 2013). Interestingly, OsDRM2 was found to interact with the ATP-dependent RNA helicase, OseIF4A, in both *in vitro* and *in vivo* assays (Dangwal et al., 2013). The interaction specifically depends on the ubiquitin-associated domain of OsDRM2, pointing to a mechanism in which OsDRM2 is recruited to specific chromatin sites by eIF4A together with other cellular proteins for catalyzing DNA methylation (Dangwal et al., 2013). Similar to the *Arabidopsis DECREASE IN DNA METHYLATION 1* (*DDM1*), which encodes a nucleosome remodeling ATPase, *OsDDM1* is also necessary for maintenance of DNA methylation in transposons and repetitive sequences (Higo et al., 2012). The rice genome contains three putative CMT3 homologs (Sharma et al., 2009), yet their functions remain to be characterized.

DNA methylation can be removed passively through dilution during replication as well as actively through catalysis by demethylation enzymes (La et al., 2011; Ono et al., 2012). In *Arabidopsis*, active demethylation is catalyzed by REPRESSOR OF SILENCING 1 (ROS1; Gong et al., 2002; Agius et al., 2006), DEMETER (DME; Choi et al., 2002; Gehring et al., 2006), and DEMETER-LIKE 2 (DML2) and DML3 (Choi et al., 2002; Ortega-Galisteo et al., 2008). Phylogenetic analysis showed that the rice genome encodes six putative bi-functional DNA glycosylases that catalyze cytosine DNA demethylation: four ROS1 orthologs (ROS1a to ROS1d) and two DML3 orthologs (DML3a and DML3b), but no DME orthologs (Zemach et al., 2010). *ROS1c* has been shown to be involved in DNA demethylation and control of the retrotransposon *Tos17* activity (La et al., 2011). Quantitative RT-PCR analysis revealed that *ROS1a*, *ROS1d,* and *DML3a* are expressed in different examined plant tissues, including anthers and pistils, whereas *ROS1b* and *DML3b* are scarcely expressed in these tissues (Ono et al., 2012). Future studies are necessary to investigate the role of these different genes in rice genome DNA methylation.

### HISTONE METHYLATION

Histone methylation marks are established on lysine (K) and arginine (R) residues by distinct enzymes, namely histone lysine methyltransferases (HKMTs) and protein arginine methyltransferases (PRMTs), respectively (Liu et al., 2010; Yao and Shen, 2011). In general, H3K9, H3K27, and H4K20 methylations are associated with transcriptional repression, whereas methylation on H3K4 and H3K36 correlates with gene activation. Furthermore, each K residue can be mono-, di-, or tri-methylated, and different methylation status may have different functional implications (Yu et al., 2009).

All known plant HKMTs contain an evolutionarily conserved SET domain (reviewed in Berr et al., 2011). The rice genome encodes at least 37 SET domain proteins, grouped into distinct families (Ng et al., 2007; Huang et al., 2011; Thorstensen et al., 2011). To date, several members belonging to different families are characterized (**Table 1**). Analyses of SET DOMAIN GROUP 714 (SDG714) and its close homologs (e.g., SDG728) showed that these rice SDG proteins have either specific or redundant functions in regulating histone H3K9 methylation and retrotransposon repression (Ding et al., 2007a,b, 2010; Qin et al., 2010). Knockdown of *SDG714* leads to decreased H3K9 methylation levels accompanied by a reduction of CG and CHG methylation, suggesting that H3K9 methylation and DNA methylation act closely together to stably repress the transposition of transposons to maintain genome stability (Ding et al., 2007b). Ectopic expression of *SDG714* in *Arabidopsis* can cause a global elevation of H3K9me2 (Ding et al., 2010). Knockdown of *SDG725* impairs deposition of H3K36me2/3 at several examined gene loci (Sui et al., 2012, 2013). SDG724 is also involved in H3K36me2/3 deposition (Sun et al., 2012). SDG723/OsTrx1 is a close homolog of the *Arabidopsis* H3K4-methyltransferase ATX1 and can methylate *in vitro* H3 within oligonucleosomes (Choi et al., 2014). The rice genome contains two genes encoding putative H3K27 methyltransferases, OsiEZ1/SDG718 (also named OsSET1) and OsCLF/SDG711, which likely work in protein complexes in Polycomb silencing pathway (see Section below).

Histone lysine methylation can be removed by histone demethylases, which consist of two classes: Lysine Specific Demethylase 1 (LSD1) and Jumonji C (jmjC) domain-containing proteins (Tsukada et al., 2006; Mosammaparast and Shi, 2010). LSD1, a flavin-dependent amine oxidase, has been the first histone demethylase reported (Shi et al., 2004) and *Arabidopsis* contains three LSD1 homologs, which are involved in flowering time regulation (Jiang et al., 2007; Liu et al., 2007; Shafiq et al., 2014). Three rice genes (*Os02g0755200*, *Os04g0560300,* and *Os08g0143400*) encode LSD1 homologs, but their functions remain uncharacterized. There are at least 20 jmjC domain-containing proteins in rice, and the first characterized JMJ706 specifically demethylates H3K9me2/me3 (Sun and Zhou, 2008). More recently, several other rice jmjC-encoding genes have been characterized. *JMJ705* encodes a histone lysine demethylase that specifically removes H3K27me2/3, and the expression of *JMJ705* is induced by stress signals and during pathogen infection (Li et al., 2013). For active histone marks, JMJ703 is involved in the removal of H3K4me1/me2/me3 (Chen et al., 2013; Cui et al., 2013), and JMJ701 in removal of H3K4me3 (Yokoo et al., 2014). So far, however, histone demethylase(s) involved in removal of H3K36 methylation is(are) unknown.

### POLYCOMB SILENCING

Polycomb Group (PcG) proteins were first identified as master regulators and suppressors of homeotic genes in *Drosophila melanogaster*. Polycomb Repressive Complex 2 (PRC2) has four core components: ENHANCEROF ZESTE (E[z]), SUPPRESSOR OF ZESTE 12 (Su[z]12), EXTRA SEX COMBS (ESC), and the 55 kDa WD40-repeat protein N55 (Schuettengruber and Cavalli, 2009). PRC2 mediates H3K27me3 deposition *via* the catalytic subunit E[z], a SET-domain containing protein (Czermin et al., 2002). The four core subunits of the PRC2 complex are well conserved in animals as well as in plants (Chen and Rasmuson-Lestander, 2009; He et al., 2013). While in *Drosophila* all but one subunit is encoded by a single gene, most of the plant PRC2 core subunits are encoded by small gene families. In *Arabidopsis*, MEDEA (MEA)/FERTILIZATION INDEPENDENT SEED 1 (FIS1), CURLY LEAF (CLF), and SWINGER (SWN) are the three homologs of E[z]; FIS2, VERNALIZATION 2 (VRN2), and EMBRYONIC FLOWER 2 (EMF2) are the three homologs of Su[z]12; MULTICOPY SUPPRESSOR OF IRA1 (MSI1) to MSI5 are the five homologs of N55; and FERTILIZATION INDEPENDENT ENDOSPERM (FIE) is the only homolog of ESC. Remarkably, MEA/FIS1 and FIS2, which are important for endosperm and seed development in *Arabidopsis*, are absent from rice, and rice has two E[z] homologs: OsiEZ1/SDG718 and OsCLF/SDG711, two Su[z]12 homologs: OsEMF2a and OsEMF2b, but also two FIE homologs: OsFIE1 and OsFIE2 (Luo et al., 2009). Functional roles of some of these rice PcG proteins have been characterized (**Table 1**). The expression of *OsiEZ1/SDG718* and *OsCLF/SDG711* is induced by and represses flowering genes in long day and short day, respectively (Liu et al., 2014). While *OsFIE2* is expressed broadly in all examined rice tissues, *OsFIE1* is expressed specifically in the rice endosperm and its expression in vegetative tissues is likely to be silenced by promoter DNA methylation (Zhang et al., 2012b; Nallamilli et al., 2013). Furthermore, *OsFIE1* is imprinted and only the maternal allele is expressed in endosperm (Luo et al., 2009). More recently, it was reported that *OsFIE1* is responsive to temperature changes and its expression negatively correlates with the duration of the syncytial seed developmental stage during heat stress (Folsom et al., 2014). DNA methylation, H3K9me2 and/or H3K27me3 are likely involved in regulation of varied repressive status of *OsFIE1* (Zhang et al., 2012b; Nallamilli et al., 2013; Folsom et al., 2014). Functional characterization of *OsEMF2b* revealed that PRC2 plays a major role in modulation of the expression of E-function MADS-box transcription factor genes required for floral organ specification and floral meristem determinacy (Luo et al., 2009; Yang et al., 2013; Conrad et al., 2014). Very importantly, OsFIE2 interacts with OsiEZ1/SDG718 and the OsFIE2-associated complex purified from transgenic rice suspension cells (containing OsEMF2b, OsCLF, OsiEZ1/SDG718) can methylate H3K27 in *in vitro* histone methyltransferase assay (Nallamilli et al., 2013).

### HISTONE ACETYLATION

Histone lysine acetylation is generally associated with transcription activation and is dynamically regulated by the antagonistic activities between histone acetyltransferases (HATs) and histone deacetylases (HDACs; Chen and Tian, 2007). All four core histones can be acetylated and a nucleosome contains 26 putative acetylation sites (Lusser et al., 2001). Global analysis of lysine acetylation demonstrates the involvement of protein acetylation in diverse biological processes in rice (Nallamilli et al., 2014). The rice genome contains eight HATs and 19 HDACs (Hu et al., 2009; Liu et al., 2012). The eight HATs can be divided into four groups, namely the CREB-Binding Protein (CBP) group, the TAFII-associated factor (TAFII250) group, the GCN5-related *N*-terminal acetyltransferase (GNAT) group, and the MYST (named for the founding members MOZ, Ybf2/Sas3, Sas2, and Tip60) group (Liu et al., 2012). The 19 HDACs are grouped into three distinct families, namely the Reduced Potassium Deficiency 3 (RPD3) family, the Silent Information Regulator 2 (SIR2) family, and the type-II HDAC (HD2) family which is plant specific (Ma et al., 2013). Reversible and dynamic changes of H3 acetylation occurs at submergence-inducible genes, *alcohol dehydrogenase 1* (*ADH1*) and *pyruvate decarboxylase 1* (*PDC1*) in rice (Tsuji et al., 2006). Forward genetic analysis has identified a rice mutant, *rice plasticity 1* (*rpl1*), which displays increased environment-dependent phenotypic variations and an elevation of overall H3K9 acetylation (Zhang et al., 2012a). Down-regulation of *OsHDT1/HDT701*, which encodes a histone H4 deacetylase, causes elevated levels of H4 acetylation and increased transcription of pattern recognition receptor (PRR) and defense-related genes (Ding et al., 2012a). Knockdown of *OsSRT1*, a member of SIR2-like HDAC family, results in an increase of H3K9 acetylation (H3K9ac), leading to DNA fragmentation and cell death, and the OsSRT1 protein binds to loci with relative low level of H3K9ac and regulates expression of many genes related to stress and metabolism as well as several families of transposable elements (Huang et al., 2007; Zhong et al., 2013).

### READERS OF HISTONE MODIFICATIONS

Specific recognition of histone modifications by readers can recruit various components of the nuclear signaling network to chromatin, mediating fundamental processes such as gene transcription, DNA replication and recombination, DNA repair and chromatin remodeling (Musselman et al., 2012). Some readers are reported in *Arabidopsis* (reviewed in Berr et al., 2011), and more recent works have identified several novel chromodomain (CHD)- and/or plant homeodomain (PHD)-containing proteins as readers of H3K4me2/me3 and H3K36me3 (Bu et al., 2014; Lopez-Gonzalez et al., 2014; Molitor et al., 2014; Xu et al., 2014). Interestingly, the rice CHD3 protein can bind both the active mark H3K4me2 and the repressive mark H3K27me3 via its CHD and PHD domain, respectively (Hu et al., 2012). Knockdown of CHD3 caused reduction of H3K4me3 and H3K27me3 at many genes. It was thus suggested that the rice CHD3 may act as a bifunctional reader capable to recognize and modulate both H3K4 and H3K27 methylations (Hu et al., 2012).

### SMALL AND LONG NON-CODING RNAs

Non-coding small RNAs (sRNA) of 21–24 nucleotides (nt) in length as well as long non-coding RNAs (lncRNAs, >200 nt in length) are known to be involved in chromatin modifications and thus epigenetic inheritance (reviewed in Castel and Martienssen, 2013; Bond and Baulcombe, 2014). Genome-wide profiling have identified several hundreds of different sRNAs, and differences exist at their expression levels between different rice subspecies, reciprocal hybrids, different plant tissues, and under different growth conditions (Chen et al., 2010; He et al., 2010; Jeong et al., 2010; Zhang et al., 2014). Remarkably, the most abundant sRNAs identified in rice panicles are 24 nt in length and mainly correspond to transposon-associated or repeat-associated small interfering RNAs (siRNAs; Jeong et al., 2011). The most intriguing role of siRNAs is in repression of transposons and repeat elements in reproductive tissues and epigenomic reprogramming during gametogenesis (Gutierrez-Marcos and Dickinson, 2012; Castel and Martienssen, 2013; Bond and Baulcombe, 2014). ARGONAUTE (AGO) proteins play important roles in microRNA-mediated post-transcriptional gene silencing (PTGS) and siRNA-mediated RdDM (Vaucheret, 2008). A germ line specific AGO-encoding gene, *MEIOSIS ARRESTED AT LEPTOTENE1* (*MEL1*), has been reported in rice, and the *mel1* mutant shows chromosome abortion during early meiotic stages, leading to impaired male and female fertilities (Nonomura et al., 2007). More recently, forward genetic analysis has identified a lncRNA, which could be subsequently processed to small RNAs, as a key regulator of male fertility in rice (Ding et al., 2012b,c). Meanwhile, Zhou et al. (2012) reported that a spontaneous mutation of a small RNA could cause male sterility in rice. Nevertheless, the precise role of lncRNA and sRNA, particularly at rice chromatin structure levels, requires future investigations.

## EPIGENETIC REGULATION OF RICE FLOWERING

**FIGURE 1:**
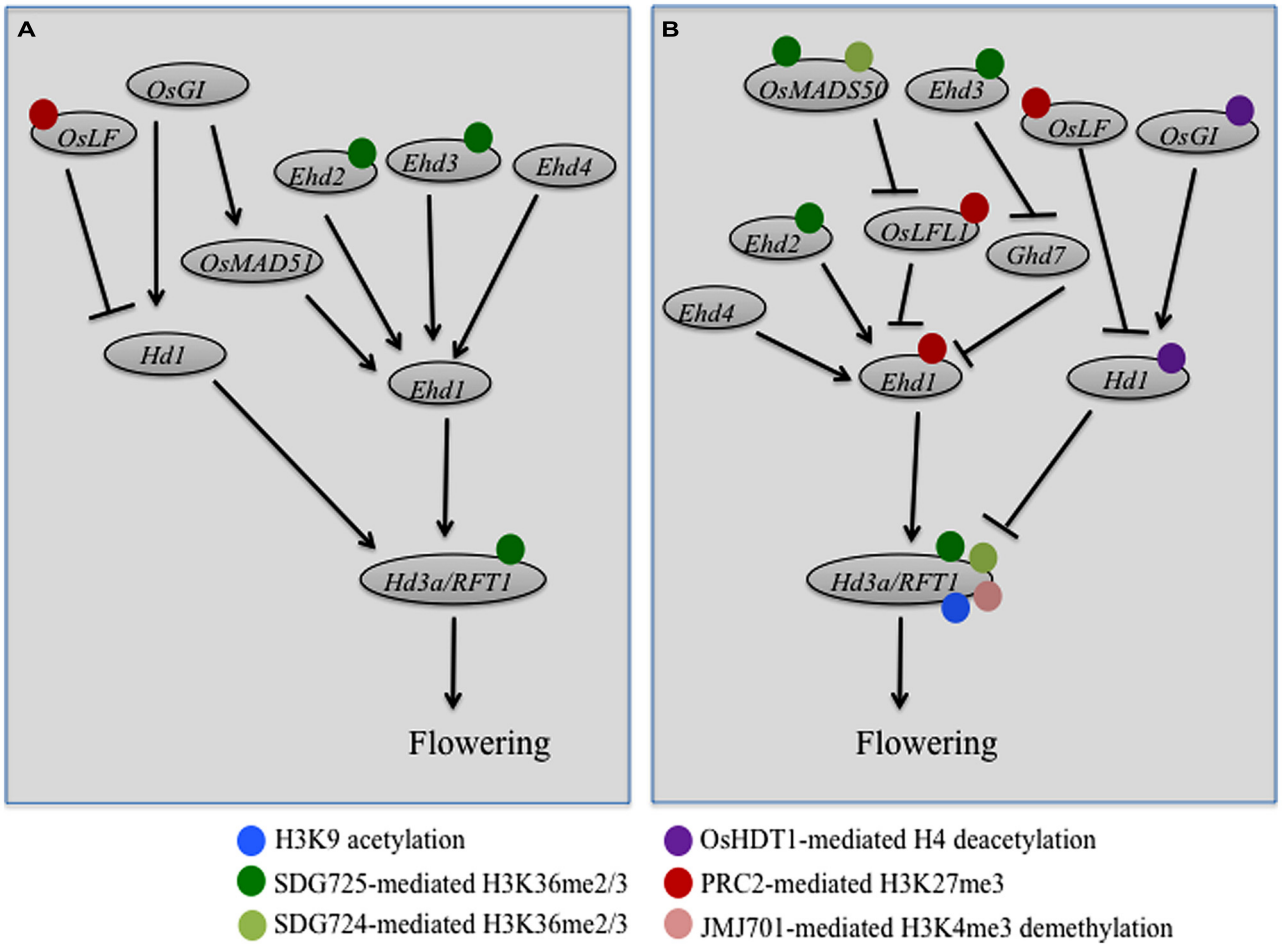
**Regulatory networks of genetic and epigenetic control of rice flowering under short-day (A) and long-day (B) photoperiod conditions.** Rice flowering network is integrated by two florigen genes *Hd3a* and *RFT1*, which are regulated by at least two pathways: the *Hd1*-dependent and the *Ehd1*-dependent pathways. Expressions of *Hd1* and *Ehd1* are further regulated by more upstream genes as indicated by different names in the circles. Arrows indicate for transcriptional activation, whereas bars indicate for transcriptional repression. Different color spheres surrounding the flowering gene circles indicate for different regulations by the indicated histone modifications at the gene locus, currently described in literatures.

Flowering represents the transition from vegetative to reproductive growth, a key developmental switch during the plant life cycle. Flowering time is precisely controlled by complex gene network that integrates environmental signals, such as day length (photoperiod), light intensity and quality, and ambient temperature, as well as endogenous cues involving plant hormones (Albani and Coupland, 2010; Shrestha et al., 2014). Photoperiod is one of the most predictable cues in nature, and according to photoperiod responsiveness plants can be categorized into three groups: long-day (LD) plants, short-day (SD) plants, and day-neutral plants. *Arabidopsis* is a facultative LD plant whose flowering is accelerated when grown under LD photoperiods. Furthermore, flowering of most *Arabidopsis* ecotypes is promoted by a prolonged exposure to the cold of winter (a process known as vernalization), which has an epigenetic basis of competence memory (Ream et al., 2012; Song et al., 2012). During recent years, many chromatin modifiers have been shown as involved in *Arabidopsis* flowering time regulation, with majority of them acting *via* the transcriptional regulation of *FLOWERING LOCUS C* (*FLC*), a key flowering repressor at which vernalization and autonomous pathways converge (Berr et al., 2011; He, 2012; Ietswaart et al., 2012). In contrast to *Arabidopsis*, rice is a facultative SD plant and does not require vernalization to induce flowering and does not contain a *FLC* homolog. The complex gene network of rice flowering pathways primarily consists of flowering activators, and remarkably several chromatin modifiers have been shown recently as involved in rice flowering time control (**Figure 1**).

### KEY TRANSCRIPTION FACTORS OF RICE FLOWERING PATHWAYS

Within the rice flowering pathways, the close paralogs *Heading date 3a* (*Hd3a*) and *RICE FLOWERING LOCUS T1* (*RFT1*) are specifically upregulated upon the inductive SD photoperiods in leaf phloem tissue and encode small globular proteins named florigens, which move to the shoot apex to promote flowering (Tsuji et al., 2013; Sun et al., 2014). There are at least two pathways that control the *Hd3a/RFT1* expression under either SD (**Figure 1A**) or LD (**Figure 1B**) photoperiods: the *Early heading date 1* (*Ehd1*) and the *Hd1* pathways (Tsuji et al., 2013; Sun et al., 2014). *Ehd1* encodes a B-type transcription factor that plays a key role in activation of both *Hd3a* and *RFT1* expression. The expression of *Ehd1* is modulated by at least three different types of function factors (Sun et al., 2014). The first type comprises day length-independent activators, including *Ehd2*, also known as *Rice Indeterminate1* (*RID1*) or *Os Indeterminate1* (*OsId1*), and *Ehd4*, which encode two different zinc-finger transcription factors and act in both SD and LD conditions in *Ehd1* induction (**Figure 1**). The second type comprises SD-preferential activators, including the PHD-finger factor Ehd3 and the MADS-box family transcription factor OsMADS51, which induce *Ehd1* expression specifically in SD conditions (**Figure 1A**). And the third type comprises LD-preferential repressors, including *Grain number, plant height, and heading date7* (*Ghd7*) that encodes a CCT-domain protein and *LEC2-FUSCA3-Like 1* (*OsLFL1*) that encodes a B3-type transcription factor, both repress *Ehd1* expression specifically in LD conditions (**Figure 1B**). Further upstream, the LD-preferential regulator *OsMADS50* promotes flowering *via* repression of *OsLFL1*. Interestingly, *Ehd3,* which acts as an activator of *Ehd1* to promote flowering in SD conditions (**Figure 1A**), displays a repressor function on *Ghd7* and thus also promotes flowering in LD conditions (**Figure 1B**). The rice circadian clock related protein GIGANTEA (OsGI) activates the *Ehd1* pathway partly *via* induction of *OsMAD51* expression (**Figure 1B**). While the *Ehd1* pathway is more unique to rice, the OsGI-Hd1-Hd3a pathway is very similar to the *Arabidopsis* GI-CO-FT pathway, composing of the respective orthologous proteins in the two plant species (Tsuji et al., 2013; Sun et al., 2014). An atypical helix-loop-helix (HLH) protein (OsLF) also is involved in the OsGI-Hd1-Hd3a pathway via *Hd1* repression. *Hd1* acts as an activator to promote rice flowering in SD conditions (**Figure 1A**) but as a suppressor of rice flowering in LD conditions (**Figure 1B**). Phytochrome signaling is crucial in conversion of Hd1 activity because mutation of *Phytochrome B* (*PHYB*) or phytochrome deficiency (e.g., in *photoperiod sensitivity5* mutant) maintains Hd1 as an activator independent of day length. Under LD conditions, the red-light photoreceptor PHYB pathway may convert and maintain Hd1 as a repressor possible *via* post-translational modification and/or protein complex formation. Because of space limitation, the one who is interested in more details about rice flowering pathways can read the two excellent review articles here cited (Tsuji et al., 2013; Sun et al., 2014) and the original research papers referred therein.

### ACTIVE CHROMATIN MARKS ARE INVOLVED IN RICE FLOWERING TIME REGULATION

Understanding how the rice flowering pathway genes are regulated in the chromatin context has great importance. Recent studies have found that histone acetylations, H3K4 and H3K36 methylations are involved in active transcription of several genes within the rice flowering pathways (**Figure 1**). It was reported that overexpression of the HD2-family HDAC gene *OsHDT1* in hybrid rice leads to early flowering under LD conditions, probably through transcriptional repression of *OsGI* and *Hd1* (Li et al., 2011a). Interestingly, the expression of *OsHDT1* displays a circadian rhythm under SD conditions, peaked at the end of day, which coincides with rhythmic expression of *OsGI* and advances that of *Hd1*. Ectopic *OsHDT1* expression in transgenic rice attenuates the overdominance rhythmic expression of *OsGI* and *Hd1* in hybrid rice, which may explains the early flowering phenotype specifically observed in hybrid but not parental rice lines (Li et al., 2011a). Histone H4 acetylation levels were observed to positively correlate with the rhythmic expression of *OsGI* and *Hd1*, and *OsHDT1* overexpression was shown to impair the acetylation increase at the peak time (Li et al., 2011a).

A positive DNA/histone methylation role in rice flowering promotion was first indicated by the study of the *S*-adenosyl-L-methionine synthetase gene mutants (Li et al., 2011b). *S*-Adenosyl-L-methionine is a universal methyl group donor for both DNA and protein methylations. Its deficiency caused late-flowering of rice plants and reduction of *Ehd1*, *Hd3a*, and *RFT1* expression, which is associated with reduced levels of H3K4me3 and DNA CG/CHG-methylations at these flowering gene loci (Li et al., 2011b). More recently, it was reported that suppression of *OsTrx1*, an ortholog of the *Arabidopsis* H3K4-methyltransferase gene *ATX1*, delays rice flowering time under LD conditions (Choi et al., 2014). The *OsTrx1* suppression did not affect the *OsMADS50* and *Hd1* pathways, but elevated *Ghd7* expression and drastically reduced *Ehd1*, *Hd3a* and *RFT1* expression, which is consistent with the plant late-flowering phenotype (**Figure 1B**). The PHD domain of OsTrx1 can bind to native histone H3 and the SET domain of OsTrx1 can methylate histone H3 from oligonucleosomes *in vitro* (Choi et al., 2014). Yet the role of OsTrx1 in histone methylation *in vivo* remains undemonstrated. Because the OsTrx1 and Ehd3 proteins bind each other, the authors propose that OsTrx1 may promote rice flowering *via* interaction with Ehd3 (Choi et al., 2014). Mutant characterization of *Photoperiod sensitivity-14*(*Se14*), which encodes the JmjC-domain protein JMJ701, revealed that H3K4me3 elevation at the *RFT1* promoter region increases *RFT1* expression, leading to rice plant early flowering under LD conditions (Yokoo et al., 2014). It is currently unknown whether or not OsTrx1 and JMJ701 could work as a couple in an antagonistic manner to control H3K4me3 levels at the *RFT1* locus.

H3K36me3 is generally considered as acting more downstream of H3K4me3 during transcription processes (Berr et al., 2011). The first H3K36-methyltransferase characterized in rice is SDG725, which has been shown to specifically methylate H3K36 from mononucleosomes *in vitro* and is required for H3K36me2/me3 deposition at chromatin regions of genes related to brassinosteroid biosynthesis or signaling pathways (Sui et al., 2012). Knockdown of *SDG725* caused a rice plant late-flowering phenotype (Sui et al., 2012), and subsequent investigation revealed that SDG725 is necessary for H3K36me2/3 deposition at several flowering genes including *Ehd3*, *Ehd2*, *OsMADS50*, *Hd3a*, and *RFT1* (Sui et al., 2013). Characterization of the late-flowering mutant named *long vegetative phase 1* (*lvp1*) together with map-based cloning has uncovered *SDG724* as an essential regulator of the *OsMAD50*-*Ehd1*-*RFT1* pathway (Sun et al., 2012). The recombinant SDG724 protein can methylate H3 (with K site undetermined) from oligonucleosomes and the *lvp1* mutant plants show global reduction of H3K36me2/me3 levels. Remarkably, ChIP analysis revealed specific reduction of H3K36me2/me3 at *OsMADS50* and *RFT1* but not at *Ehd1* and *Hd3a* in the *lvp1* mutant plants (Sun et al., 2012). Both the *lvp1* (*sdg724*) mutant and the SDG725-knockdown mutant exhibit late-flowering phenotypes under either SD or LD conditions (Sun et al., 2012; Sui et al., 2013), pointing to a crucial role of H3K36me2/me3 in promoting rice plant flowering irrespective of photoperiods. It is noteworthy that in *Arabidopsis* the SDG8-mediated H3K36me2/me3 also plays a major role in flowering time control, but in that case in prevention of early flowering (Shafiq et al., 2014). Future studies are necessary to investigate mechanisms underlying the overlap and specific targets of SDG724 and SDG725 in the rice flowering time control.

### REPRESSIVE CHROMATIN MARKS ARE INVOLVED IN RICE FLOWERING TIME REGULATION

The repressive mark H3K27me3 is known to play a key role in *FLC* repression in vernalization-induced *Arabidopsis* plant flowering (He, 2012; Ietswaart et al., 2012). Interestingly, recent studies have shown that H3K27me3 deposited by PRC2-like complexes also plays an important role in vernalization-independent rice flowering time control (**Figure 1**). Loss-of-function of the PRC2 gene *OsEMF2b* causes late-flowering, which is associated with an increase of *OsLFL1* expression and a decrease of *Ehd1* expression (Yang et al., 2013). The OsEMF2b protein physically interacts with OsVIL3 (named as OsVIL2 in Yang et al., 2013, but here corrected to the first nomenclature used in Zhao et al., 2010; also called LC2), a PHD-domain protein showing homologies to the *Arabidopsis* VIN3-group proteins including VERNALIZATION INSENSITIVE 3 (VIN3), VIN3-LIKE 1 (VIL1)/VRN5, and VIL2/VEL1. The *Arabidopsis* VIN3-group proteins are know to be associated and to work together with the PRC2 core complex (constituting the so-called PHD-PRC2 complexes) and the *VIN3* expression is induced early during vernalization (reviewed in He, 2012; Ietswaart et al., 2012). Consistent with the idea that OsVIL3/LC2 works together with PRC2, knockdown of *OsVIL3/LC2* results in rice late-flowering, increase of *OsLFL1* and *OsLF* expression, and decrease of *Ehd1* as well as *Hd3a* and *RFT1* expression (Wang et al., 2013; Yang et al., 2013). The OsVIL3/LC2 protein binds at the *OsLFL1* and *OsLF* chromatin regions and the H3K27me3 enrichments at *OsLFL1* and *OsLF* are impaired in the *osvil3/*lc2 mutant (Wang et al., 2013; Yang et al., 2013). In addition to *OsVIL3/LC2*, *OsVIL2* plays a similar but non-redundant role in rice flowering time control. Expression of both *OsVIL3/LC2* and *OsVIL2* is induced by SD conditions and the OsVIL3/LC2 and OsVIL2 proteins physically interact, thus leading to the proposition that the OsVIN3/LC2-OsVIL2 dimer may recruit PRC2 in H3K27me3 deposition and *OsLF* suppression in rice photoperiod flowering regulation (Wang et al., 2013). Very recently, *OsiEZ1/SDG718* and *OsCLF/SDG711* have been reported to display distinct roles in photoperiod regulation of flowering (Liu et al., 2014). While *OsiEZ1/SDG718* is induced in SD conditions and represses *OsLF* to promote flowering (**Figure 1A**), *OsCLF/SDG711* is induced in LD conditions and represses *OsLF* and *Ehd1* to inhibit flowering (**Figure 1B**). The OsCLF/SDG711 protein has been shown to target *OsLF* and *Ehd1* loci to mediate H3K27me3 deposition and gene repression (Liu et al., 2014).

## EPIGENETIC REGULATION OF RICE REPRODUCTION AND SEED FORMATION

**FIGURE 2:**
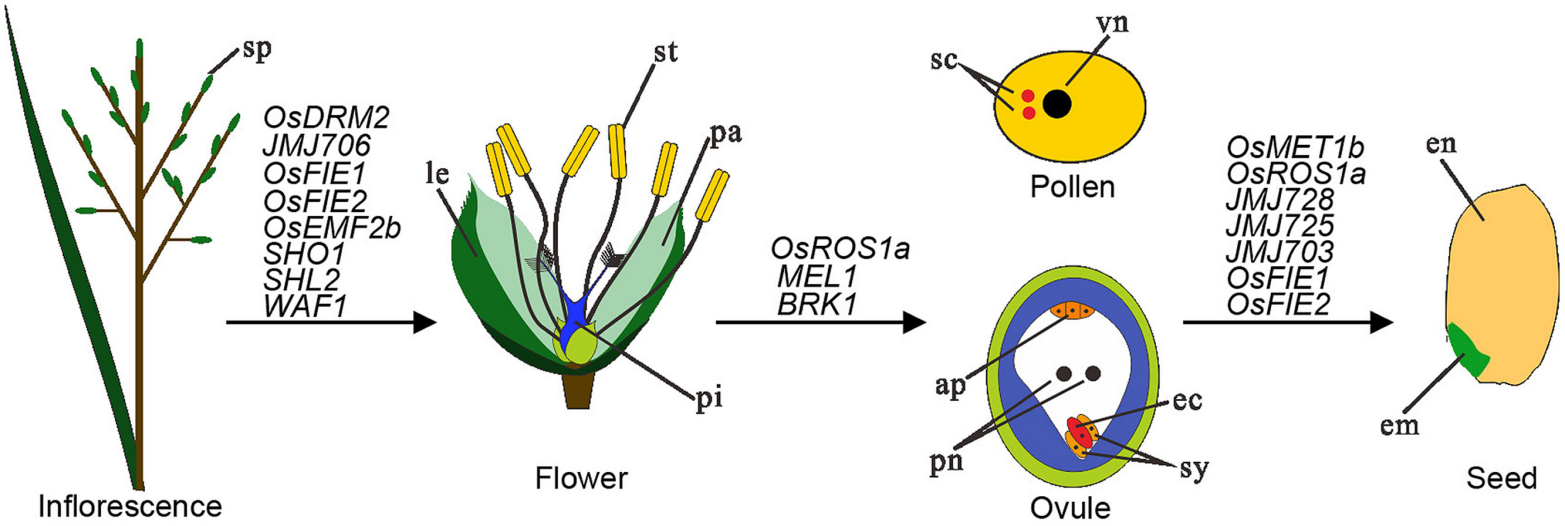
**Schematic representation of structures involved in rice reproduction together with chromatin modifier genes listed in regulation of three different steps.** Inflorescence produces spikelets (sp) that generate numerous flowers. A mature flower contains different types of organs including lemma (le), palea (pa), stemen (st), and pistil (pi). The female gametophyte ovule is formed inside of ovary of the pistil and at maturation contains four different types of cells: antipodal (ap), polar nuclei (pn), synergid (sy), and egg cell (ec). The male gametophyte pollen is produced inside of anther of the stamen and at maturation contains two sperm cells (sc) and one vegetative cell (vn). Upon fertilization, one sperm cell fuses with egg cell to produce embryo (em) and the other sperm cell fuses with the two polar nuclei to produce endosperm (en), together forming a mature seed. Chromatin modifier genes playing important regulatory roles in floral organogenesis, gametophyte development, and fertilization/seed development are listed.

After flowering, plant sexual reproduction occurs in dedicated floral organs through sporogenesis, gametogenesis, embryo- and endosperm-genesis, resulting in seed formation. Studies in *Arabidopsis* have unraveled diverse epigenetic regulatory mechanisms as involved in different processes during floral organogenesis and plant sexual reproduction (Shen and Xu, 2009; Engelhorn et al., 2014; She and Baroux, 2014). Although more recent, studies in rice also have started to uncover multiple types of epigenetic modifiers involved in the regulation of plant reproduction (**Figure 2**).

### EPIGENETIC REGULATION IN RICE REPRODUCTION

Compared to those of *Arabidopsis*, the rice inflorescence and flower have greatly diverged structures that are regulated by a conserved genetic framework together with rice specific genetic mechanisms (Yoshida and Nagato, 2011). Several epialleles are found to affect rice plant reproduction. The metastable epigenetic silencing of *DWARF1*, which is associated with DNA methylation and H3K9me2 at the gene promoter region, causes dwarf tillers, compact panicles (inflorescences) and small round rice grains (Miura et al., 2009). The *abnormal floral organ* (*afo*) epimutation causes increased DNA methylation and suppression of the transcription factor gene *OsMADS1*, leading to pseudovivipary, a specific asexual reproductive strategy (Wang et al., 2010). The transcription factor gene *SQUAMOSA PROMOTER BINDING PROTEIN-LIKE 14* (*SPL14*), also known as *IDEAL PLANT ARCHITECTURE 1* (*IPA1*) or *WEALTHY FARMER’S PANICLE* (*WFP*), promotes panicle branching and regulates a large number of genes, and differences in DNA methylation at the locus as well as the micro RNA 156 (OsmiR156) contribute to expression differences of *SPL14*/*IPA1*/*WFP* in different rice varieties (Jiao et al., 2010; Miura et al., 2010; Lu et al., 2013). Important roles of sRNA (both miRNAs and siRNAs) in rice floral organ development are also evidenced by mutants of several sRNA-pathway genes, including *SHOOT ORGANIZATION 1* (*SHO1*) encoding a DICER-LIKE 4 homolog, *SHOOTLESS 2* (*SHL2*) encoding a RDR6 homolog), and *WAVY LEAF 1* (*WAF1*) encoding a HEN1 homolog (Abe et al., 2010; Toriba et al., 2010). lncRNAs are also reported as involved in plant reproductive process (Swiezewski et al., 2009; Heo and Sung, 2011). In rice, a point mutation that alter the secondary structure of the lncRNA called Long-Day-specific Male-fertility-Associated RNA (LDMAR) has been found to cause the photoperiod sensitive male sterility (Ding et al., 2012b).

Importance of DNA methylation in regulation of rice reproduction has been further supported by mutant studies. Targeted disruption of the DNA demethylase gene *ROS1a* leads to paternal allele transmission defect, presumably because of a male gametophytic defect prior to fertilization (Ono et al., 2012). Disruption of *OsDRM2* led to pleiotropic developmental defects in both vegetative and reproductive stages including semi-dwarfed stature, reductions in tiller number, and complete sterility (Moritoh et al., 2012). Consistently, transcriptome analysis of isolated rice gametes by deep sequencing indicates that *OsDRM2* is expressed in male cells but low in vegetative cells (Anderson et al., 2013).

Several modifiers of histone modifications are also critical for rice reproduction (**Figure 2**). Loss-of-function of the rice PRC2 gene *OsEMF2b* results in complete sterility, and severe floral organ defects and indeterminacy that resemble loss-of-function mutants in E-class floral organ specification genes (Conrad et al., 2014). The epimutation of *OsFIE1* (*Epi-df*) that is caused by DNA hypomethylation, reduced H3K9me2 and increased H3K4me3 at the gene locus, leads to ectopic expression of *OsFIE1*, resulting in a dwarf stature, diverse floral defects, and alteration of H3K27me3 levels at hundreds of target genes (Zhang et al., 2012b). Mutation of the H3K27-demethylase gene *JMJ705* also causes partial sterility (Li et al., 2013). The *OsFIE2* RNAi lines display pleiotropic phenotypes including vegetative and reproductive organ formation, a decreased amount of pollen grains and a high proportion of male sterility (Li et al., 2014). These studies indicate that a balanced level of H3K27me3 is critical and that either its increase or decrease can cause rice reproduction defects. The other chromatin repressive mark H3K9me2/me3 is also important because mutation of the H3K9-demethylase gene *JMJ706* impairs spikelet development, including defective floral morphology and altered organ number (Sun and Zhou, 2008). Pleiotropic defective phenotypes including panicle morphology, rachis branch and spikelet numbers have also been described for mutants of the H3K36-methyltransferase gene *SDG725* and the H3K4-demethylase gene *JMJ703* (Sui et al., 2012; Cui et al., 2013), indicating that chromatin active marks also play important function during rice reproduction.

While precise reproduction processes affected by many of the above mentioned modifiers remain to be elucidated, meiosis is found to be regulated by several epigenetic factors. The rice germline-specific AGO-family protein MEL1 binds preferentially 21 nt siRNAs derived mostly from intergenic regions (Komiya et al., 2014), and its loss-of-function impairs both sporophytic germ-cell development and meiosis (Nonomura et al., 2007). The *mel1* mutant displays aberrant vacuolation of spore mother cells, and arrested chromosome condensation at early meiosis stages. H3K9me2 distribution as well as the localization of ZEP1, a component of transverse filaments of the rice synaptonemal complex, are affected in *mel1*, indicating for a role of *MEL1* in chromatin structure organization and homologous chromosome synapsis in early meiosis (Nonomura et al., 2007; Komiya et al., 2014). Histone phosphorylation is also involved in rice meiosis process. The rice Bub1-Related Kinase 1 (BRK1) is required for H2A phosphorylation and the centromeric recruitment of SHUGOSHIN 1 (SGO1), which is likely essential for generating proper tension between the homologous kinetochores at metaphase I to facilitate the accurate segregation of homologous chromosomes at anaphase I (Wang et al., 2012).

### EPIGENETIC REGULATIONS IN SEED DEVELOPMENT

Like other angiosperms, sexual double fertilization initiates rice seed development, giving rise to two fertilization products, the embryo and the endosperm. Epigenetic mechanisms are thought to have important contribution to plant hybrid vigor (heterosis), a phenomenon referring to the increased yield and biomass of hybrid offspring relative to the parents (Chen and Zhou, 2013; Groszmann et al., 2013). In line with this idea, divers epigenetic pathways are found as involved in seed development and seed quality control (**Figure 2**).

Genome-wide analyses in rice have revealed that sRNA expression, DNA methylation, and histone modifications (e.g., H3K9ac, H3K4me3, and H3K27me3) significantly differ between hybrids and their parents (He et al., 2010; Chodavarapu et al., 2012; Zhang et al., 2014). Remarkably, the amount of 24 nt siRNAs, with most of them likely involved in regulation of the starch and sucrose biosynthesis pathway, declines with the process of rice grain-filling and this decline is to a lower degree in inferior grains then superior grains (Peng et al., 2013). The siRNAs may act *via* or together with DNA methylation in heterochromatin silencing. In line with this idea, the maternal loss of *ROS1a* causes failure of early stage endosperm development, leading to incomplete embryogenesis producing irregular but viable embryos that failed to complete seed dormancy (Ono et al., 2012). While the *met1a* null mutant displays a normal phenotype, the *met1b* mutant exhibits abnormal seed phenotypes, which is associated with either viviparous germination or early embryonic lethality (Hu et al., 2014; Yamauchi et al., 2014). Levels of DNA methylation in *met1b* are broadly reduced at genome-wide scale and in particular at repetitive centromeric and transposon sequences as well as at the *OsFIE1* gene locus in the embryos (Hu et al., 2014; Yamauchi et al., 2014).

*OsFIE1* is an imprinted gene in rice endosperm but the *osfie1* mutant does not display any autonomous endosperm proliferation without fertilization, differing from the *Arabidopsis fie*, *mea* and *fis* mutants that are generally recognized with an autonomous endosperm proliferation phenotype (Luo et al., 2009). Nevertheless, over-expression of *OsFIE1* causes precocious cellularization and reduced seed size, and it has been proposed that that *OsFIE1* has a role in regulating seed enlargement under heat stress (Folsom et al., 2014). In addition, *OsFIE2* has a critical role in normal endosperm development and grain-filling. Down-regulation of *OsFIE2* results in small seeds and partial loss of seed dormancy, likely because of down-regulation of genes encoding the starch synthesis rate limiting step enzymes and multiple storage proteins (Nallamilli et al., 2013). Future studies are necessary to precise similarities and differences of PRC2-mediated H3K27me3 repression mechanisms involved in seed development between *Arabidopsis* and rice.

Involvement of other histone methylation marks in seed development are also evidenced from mutant studies (**Figure 2**). Down-regulation of the H3K9-methyltransferase gene *SDG728* reduces seed size and alters seed morphology (Qin et al., 2010). Loss-of-function of the H3K4-demethylase gene *JMJ703* causes abnormal grain phenotypes, including reduced length, width, and thickness (Cui et al., 2013). Also, knockdown of the H3K36-methyltransferase gene SDG725 results in small seed size and reduced seed weight (Sui et al., 2012).

## CONCLUSION REMARKS

The availability of full genome sequences and diverse improved powerful genomic and analytic tools have greatly advanced our knowledge about rice epigenetic modifiers and their biological roles. There are still a large number of modifiers uncharacterized, and molecular mechanisms of function of many chromatin modifiers remain to be investigated into details. It remains to be uncovered how the general histone modification and DNA methylation enzymes exert specific functions in plant growth and developmental processes and what effectors are involved. In particular, H3K27me3 is recognized as a crucial epigenetic mark associated with gene transcriptional repression, and the classical model proposes a sequential mode of action of the two Polycomb complexes: PRC2 is responsible H3K27me3 establishment, and PRC1 recognizes the H3K27me3 mark and further catalyzed downstream H2A monoubiquitination. While PRC1-like components and histone monoubiquitination have been recently studied in *Arabidopsis* (reviewed in Molitor and Shen, 2013; Feng and Shen, 2014), effectors acting together with H3K27me3 in rice remain unknown so far. Utilization of advanced technologies in proteomics, deep sequencing, and gene knockdown will facilitate future studies in functional characterization of interesting genes, investigation of protein complex composition and function, and gene networks controlling rice flowering and reproduction. The extensive agriculture breading has greatly enriched the rice germplasm resources with large collections of cultivated rice and their wild relatives. Comparative studies of different rice varieties and hybrids will likely impact on knowledge of genetics, epigenetics, and inheritance of agriculture traits as well as fundamental understanding of conservation and diversification of molecular mechanisms.

## Conflict of Interest Statement

The authors declare that the research was conducted in the absence of any commercial or financial relationships that could be construed as a potential conflict of interest.
